# The Microbiome Stress Project: Toward a Global Meta-Analysis of Environmental Stressors and Their Effects on Microbial Communities

**DOI:** 10.3389/fmicb.2018.03272

**Published:** 2019-01-10

**Authors:** Jennifer D. Rocca, Marie Simonin, Joanna R. Blaszczak, Jessica G. Ernakovich, Sean M. Gibbons, Firas S. Midani, Alex D. Washburne

**Affiliations:** ^1^Department of Biology, Duke University, Durham, NC, United States; ^2^Flathead Lake Biological Station, University of Montana, Polson, MT, United States; ^3^Department of Natural Resources and the Environment, University of New Hampshire, Durham, NH, United States; ^4^Institute for Systems Biology, Seattle, WA, United States; ^5^Molecular and Cellular Biology Program, University of Washington, Seattle, WA, United States; ^6^eScience Institute, University of Washington, Seattle, WA, United States; ^7^Center for Genomic and Computational Biology, Duke University, Durham, NC, United States; ^8^Department of Microbiology and Immunology, Montana State University, Bozeman, MT, United States

**Keywords:** diversity, global change, stability, 16S rRNA, bacteria, disturbance, phylofactor, community resistance

## Abstract

Microbial community structure is highly sensitive to natural (e.g., drought, temperature, fire) and anthropogenic (e.g., heavy metal exposure, land-use change) stressors. However, despite an immense amount of data generated, systematic, cross-environment analyses of microbiome responses to multiple disturbances are lacking. Here, we present the Microbiome Stress Project, an open-access database of environmental and host-associated 16S rRNA amplicon sequencing studies collected to facilitate cross-study analyses of microbiome responses to stressors. This database will comprise published and unpublished datasets re-processed from the raw sequences into exact sequence variants using our standardized computational pipeline. Our database will provide insight into general response patterns of microbiome diversity, structure, and stability to environmental stressors. It will also enable the identification of cross-study associations between single or multiple stressors and specific microbial clades. Here, we present a proof-of-concept meta-analysis of 606 microbiomes (from nine studies) to assess microbial community responses to: (1) one stressor in one environment: soil warming across a variety of soil types, (2) a range of stressors in one environment: soil microbiome responses to a comprehensive set of stressors (incl. temperature, diesel, antibiotics, land use change, drought, and heavy metals), (3) one stressor across a range of environments: copper exposure effects on soil, sediment, activated-sludge reactors, and gut environments, and (4) the general trends of microbiome stressor responses. Overall, we found that stressor exposure significantly decreases microbiome alpha diversity and increases beta diversity (community dispersion) across a range of environments and stressor types. We observed a hump-shaped relationship between microbial community resistance to stressors (i.e., the average pairwise similarity score between the control and stressed communities) and alpha diversity. We used Phylofactor to identify microbial clades and individual taxa as potential bioindicators of copper contamination across different environments. Using standardized computational and statistical methods, the Microbiome Stress Project will leverage thousands of existing datasets to build a general framework for how microbial communities respond to environmental stress.

## Introduction

In the past decade, the advent of high-throughput sequencing technologies has enabled microbial ecologists to characterize microbial community responses to environmental change at an unprecedented pace. Thousands of studies are now available on the impact of natural and anthropogenic stressors in controlled conditions or along environmental gradients spanning a wide range of biomes. Large collaborative endeavors, like the Earth and Human Microbiome Projects, revealed fundamental biogeographic patterns of microbial diversity under “baseline” or “steady state” conditions (Human Microbiome Project Consortium, 2012; Gilbert et al., 2014; Lloyd-Price et al., 2017; Thompson et al., 2017). While this baseline knowledge is crucial, similar large-scale initiatives are necessary for clarifying how microbiomes respond to fluctuating environmental conditions.

Environmental stressors occur over varying magnitudes, frequencies, and durations (Bender et al., 1984), introducing spatiotemporal heterogeneity into the environment. Spatiotemporal heterogeneity is a key driver in both the maintenance and depletion of biodiversity (Connell, 1978; Huston, 1979; Crain et al., 2008; Shade et al., 2012; Piggott et al., 2015). In ecology, *stressor, disturbance, perturbation*, and *threat* are often used interchangeably and refer to a variety of environmental changes (natural, anthropogenic, abiotic or biotic). Here, we use the term stressor to refer to any factor that alters steady-state environmental conditions (biotic or abiotic) and influences the growth or mortality of organisms in a community, resulting in either deterministic or stochastic shifts in stationary relative abundance profiles of microbiomes.

The consequences of environmental stressors on organisms are highly context dependent. Often stressors of increasing intensity/duration induce increasing stress levels but they can also affect organisms non-monotonically. For instance, moderate levels of a stressor, such as exposure to some chemical elements (e.g., micronutrients), can be beneficial to organisms, but extremely high levels can impose adverse effects. This common dose-response pattern is often referred to as hormesis or a subsidy-stress response (Odum et al., 1979; Odum, 1985; Calabrese and Baldwin, 2002). Those direct effects of stressors can propagate through ecological interaction webs causing collateral damage. Thus, stressors have both direct (Schimel et al., 2007) and indirect (Vellend, 2010; Evans and Wallenstein, 2014; Knelman et al., 2014) effects on individual taxa, leading to structural shifts in the microbial communities. Stressor-induced changes in community structure and diversity can, in turn, create feedbacks that further alter the host state (Reese et al., 2018), environmental conditions (Gibbons et al., 2016; Ratzke and Gore, 2018), or microbial ecosystem function (Bissett et al., 2013; Philippot et al., 2013).

In addition to ecological feedbacks, evolutionary feedbacks can influence how microbial communities respond to a stressor (Sanchez and Gore, 2013). For example, rapid evolution of antibiotic resistance can allow species to expand into environments that would normally be restrictive (Baym et al., 2016). However, despite the rapid expansion of research on environmental and host-associated microbiomes, we still have few generalizable insights for how microorganisms respond to stressors at the individual, population, or community levels (Treseder, 2008; Shade et al., 2012; Holden and Treseder, 2013; Duvallet, 2018). Taken together, the combined ecological data from each independent microbiome study could be a powerful resource for characterizing the processes underlying microbial community assembly and microbial population sensitivity or tolerance to environmental stressors. Pioneering meta-analyses focused on environmental perturbations in soil (Ramirez et al., 2018), human gut ecosystems (Duvallet et al., 2017; Gibbons et al., 2018; Jackson et al., 2018), and even across distinct environments (Shade et al., 2013) now provide valuable frameworks for cross-study analyses of 16S rRNA gene amplicon datasets. However, large-scale disturbance meta-analyses that integrate *both* host-associated and free-living microbiomes are currently lacking.

Here, we present the Microbiome Stress Project, a publicly available database of environmental and host-associated amplicon sequencing studies designed to facilitate our understanding of microbial community responses to disturbed environments through cross-study analyses. More specifically, our goal is to address the following research objectives:

1. One stressor in one environment: How consistent are microbiome responses to the same stressor within the same environment?
2. A range of stressors within an environment: Are there common microbiome responses to different stressors within the same environment?
3. One stressor across environments: Does a given stressor impose consistent effects on microbiomes across multiple environments, including host-associated and free-living systems?
4. General trends of microbiome stressor responses: Are there general impacts of stressors on microbiomes across all environments?

**Figure 1:**
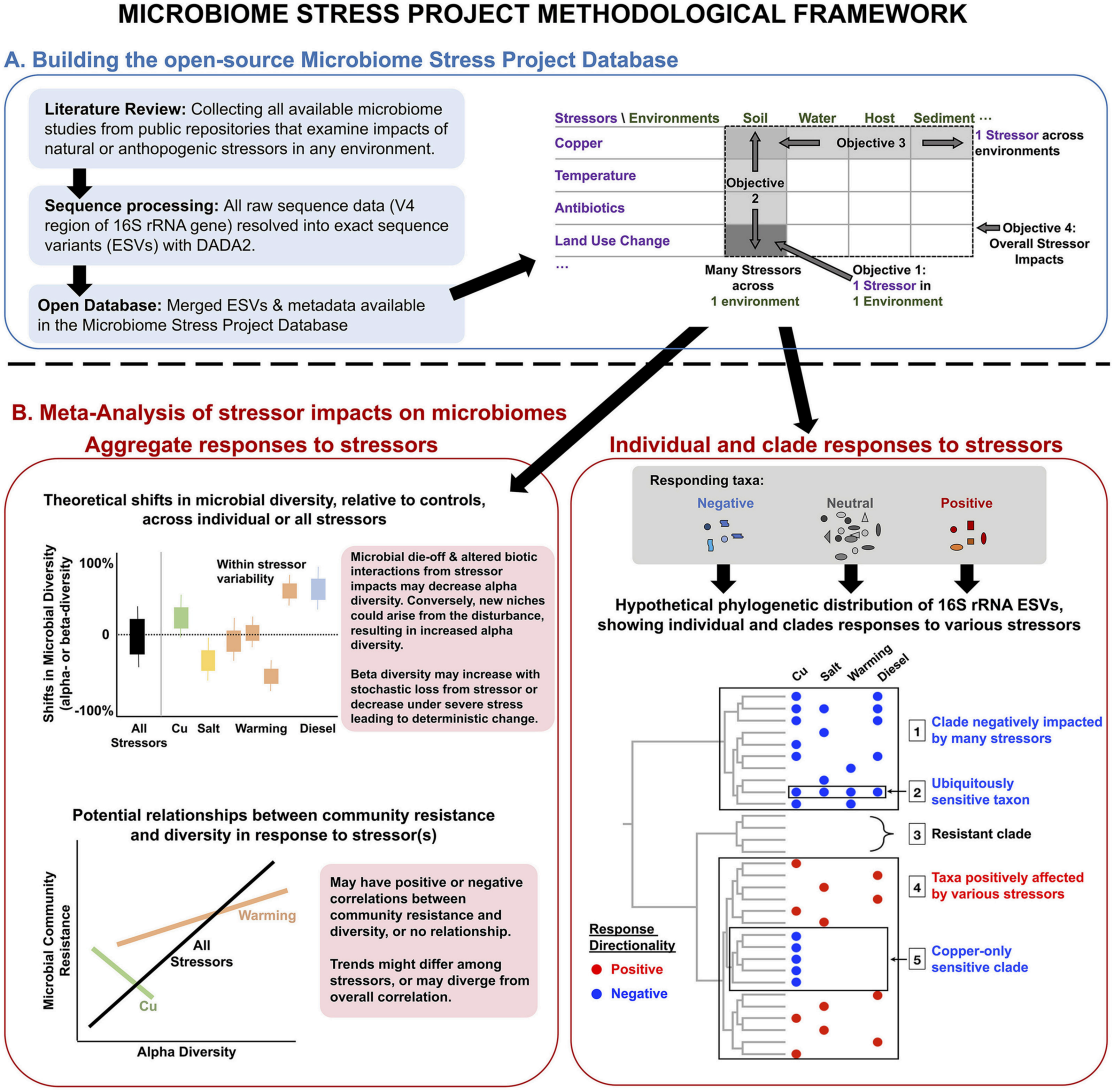
Conceptual overview of the methodological framework of the Microbiome Stress Project: from **(A)** the development of a publicly available database comprised of standardized microbial community datasets, to **(B)** the examination of wholesale impacts of stressors on microbiome diversity, and the more targeted identification of putative indicator microbial taxa or clades to a comprehensive range of stressors across multiple environments. “*Cu” stands for copper*.

These objectives lie on four principal axes for how environmental stressors impact microbiomes (Figure 1A). Capturing the variability in microbial response to a given stressor in one environment type (e.g., soil) will generate a more detailed understanding of how specific environmental parameters (e.g., soil pH) influence microbiome responses to stressors. We can also determine how the absolute magnitude of stressor treatments may be either dampened through historical exposure or exacerbated by temporal treatment regime (i.e., acute vs. chronic). Examining many stressors within the same environment enables the classification of the stressors themselves in terms of the responses they elicit, which may or may not reflect our assumptions. For instance, two stressors from the same “category” (e.g., two heavy metals) may impose similar impacts on microbiomes, but similar responses may also be possible with two distinct stressors (e.g., water stress and metal contaminations both leading to severe oxidative stress). Conversely, studying just one stressor across many environments allows us to assess the consistencies in the responses among these distinct microbiomes and to identify reliable microbial indicator taxa or clades of specific or multiple disturbances. Finally, by examining all available microbiome datasets, we can identify general effects of stressors on microbial community diversity and structure.

**Figure 2:**
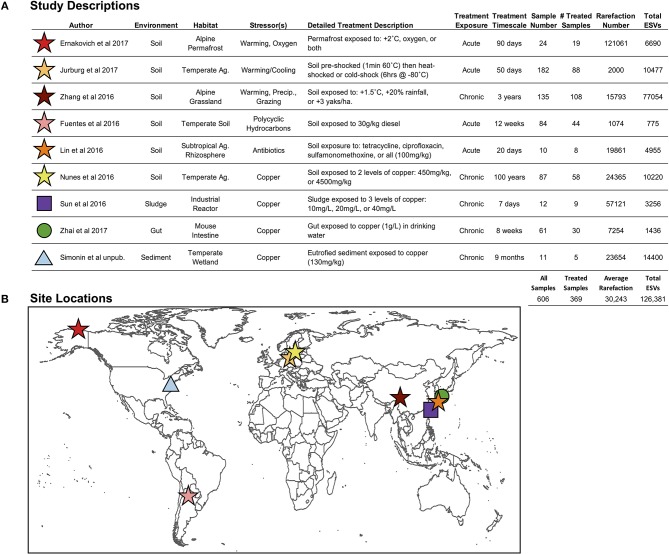
**(A)** Descriptions of environment and stressor treatments for each study included in the pilot study, and **(B)** global locations for the nine studies included in this pilot study. “*Ag.” stands for agriculture, and “Precip.” Stands for precipitation* (Fuentes et al., 2016; Lin et al., 2016; Nunes et al., 2016; Sun et al., 2016; Zhang et al., 2016; Ernakovich et al., 2017; Jurburg et al., 2017; Zhai et al., 2017).

In this article, we introduce the Microbiome Stress Project methodological framework for building and analyzing the amplicon sequencing database (Figure 1) and present the results of a proof-of-concept meta-analysis (called pilot study hereafter) using raw sequence data from a subset of the larger database (Figure 2). We start by describing the results of a literature search, identifying studies explicitly focused on the impacts of stressors on microbial communities, from which we are building the full Microbiome Stress Project Database. Then, we present the analysis pipeline used to re-process the raw amplicon sequence data in a standardized fashion for the subsequent meta-analyses. The processed data, in the form of exact sequence variant (ESV) tables and corresponding meta-data, is accessible on our website (microbiomestressproject.weebly.com), which hosts the database that will continue to grow as we add studies. The database encompasses both published and unpublished datasets, including *in situ* environmental gradients and controlled experiments, with acute (single) or chronic (repeated) stressor treatments, with either pulse (short-term) or press (long-term) exposure to the stressors. Finally, we present results from our pilot study where we examine the response of soil microbial communities to a wide set of stressors: copper, temperature, antibiotics, oxygen, polycyclic aromatic hydrocarbons, and land use change; and compare the impact of copper contamination on bacterial community structure across a range of environments: soil, gut, sediment, and activated-sludge reactors. We identify community-level responses with alpha and beta diversity, and we highlight individual- and lineage-level responses to stressors using phylofactorization (Washburne et al., 2017).

The Microbiome Stress Project is still growing and is at an early stage. Here, we present our methodological framework to demonstrate how these data can be used to understand how stressors shape microbial ecosystems. We invite the extended microbiome research community to contribute to this collaborative project and help us improve this publicly available database by sharing their data.

## Methods

### Literature Search

We performed an extensive literature search to assess the state of current research on the effects of stressors on microbial community structure. Our target questions were: (a) Which microbiome stressors are most commonly studied? (b) In which environments are these studies most commonly performed? (c) Which primers and sequencing platforms are most commonly used? and (d) How much of the microbiome sequencing data is publicly available?

A literature search was performed on Web of Science (Core Collection) in April 2018 using a comprehensive set of basic microbiome keywords (full list of keywords in Supplementary Material Table S1). We constrained the search to studies published between 2010 and 2018, which is the period of time when high-throughput sequencing methods are standardized and commonly used to measure microbial community structure and composition. We narrowed our initial search results to identify potential studies for the Microbiome Stress Project Database as those which had the basic microbiome keywords, in addition to at least one of our specific subcategory stressor keywords. We used these keywords to identify which categories of stressors (*n* = 16, e.g., heavy metals, fire, pathogens, etc.) were studied and in which environment (*n* = 11, e.g., soil, gut, reactor, etc.). These categories enabled us to address the first (a) and second (b) questions about the research landscape and determine whether or not the number of studies available is sufficient to perform robust and balanced meta-analyses.

We performed a bibliometric network analysis on the articles identified as potential candidates for the database using the unified VOS mapping technique (VOS viewer software; Van Eck and Waltman, 2010) and clustering (Waltman et al., 2010). In these networks, we investigated where the microbiome stressor studies were published and the co-citations between these journals to determine the disciplines covered by our literature search and their interactions. We also created a network based on the co-occurrence of stressor terms that were most frequently mentioned in the microbiome studies.

We investigated primer and sequencing platform usage and data availability (questions c and d) using a random subset of 150 studies, as each study needed to be manually screened for the necessary information since Web of Science keyword searches do not extract this information (i.e., this methodological information is not commonly included in abstracts or keywords). The information gleaned from question (c) was essential for standardizing the database and for developing our analytical workflow.

### Selection of Studies for the Pilot Study

We selected nine studies (606 samples; 1 independent sample = 1 microbiome) with publicly available Illumina MiSeq V4 hypervariable region 16S rRNA gene sequencing data (Figure 2; Fuentes et al., 2016; Lin et al., 2016; Nunes et al., 2016; Sun et al., 2016; Zhang et al., 2016; Ernakovich et al., 2017; Jurburg et al., 2017; Zhai et al., 2017), which were all controlled experiments that included control and treated microbiomes, enabling the calculation of proportional shifts in microbial diversity (see below section Downstream Analyses: Finding Ecological Patterns Hidden in ESVs Across Studies). These nine studies span the four axes presented in Figure 1A, targeting our primary objectives:

1. One stressor in one environment: studies restricted to soil microbiomes exposed to warming (3 studies, 79 treated microbiomes).
2. Multiple stressors within an environment: soil microbiomes exposed to 12 stressors, including exposure to: copper, temperature alteration, oxygen, antibiotics, polycyclic aromatic hydrocarbons, precipitation change, and over-grazing (6 studies, 325 treated microbiomes).
3. One stressor across environments: microbial communities exposed to copper across four environments: soil, sludge, mice gut, and sediment (4 studies, 102 treated microbiomes). The Nunes et al. (2016) study fell within objectives 2 and 3, which investigated copper exposure on soil microbiomes.
4. Overall impact of stressors on microbiomes: all microbial communities from any environment and exposed to any stressor (9 studies, 369 treated microbiomes).

### 16S rRNA Gene Sequence Processing Pipeline

Raw fastq sequence data from the 606 samples were downloaded from NCBI's Sequence Read Archive (SRA) and from an unpublished dataset, and corresponding metadata was extracted for each study via SRA file names, direct correspondence, and the published manuscript itself to identify treatment levels and treatment duration for each sample.

We chose to define phylotypes at the level of exact sequence variants (ESVs; Callahan et al., 2017). The ESV methodology identifies error-adjusted single nucleotide differences in sequence datasets to generate standardized phylotypes that can be directly compared across independently processed datasets. ESVs can be determined individually by sample or study and subsequently merged into the same feature table, allowing thousands of microbiome studies to be rapidly and efficiently combined. The ESV method provides the finest-scale taxonomic partitioning of microbial phylotypes possible and permits targeted identification of consistently-defined indicator species across datasets.

To prepare the datasets for ESV delineation, we trimmed the 16S rRNA gene sequences to the same 250 bp V4 hypervariable region within the Earth Microbiome Project primer set (515F/806R) (Gilbert et al., 2014). We used DADA2 version 1.6.0 (Callahan et al., 2017) in R version 3.5.1 (R Core Team, 2018) to resolve ESVs in each study dataset separately. DADA2 is optimized for running on separate forward and reverse fastq files. However, most of the sequence data we downloaded from SRA is only available as processed “contigs” (i.e., forward/reverse paired-ends already merged), so we proceeded with DADA2 quality control removal and dereplication on merged paired-end sequences. We acknowledge that using different algorithms to join paired ends [i.e., Qiime2 (Caporaso et al., 2010), PEAR (Kozich et al., 2013), PANDAseq (Masella et al., 2012), and UPARSE (Edgar, 2013)] may generate slightly different sequences during the joining of the forward and reverse Illumina reads due to differences in allowable mismatches and other default settings. Putative chimeras were removed with the “consensus” method using *removeBimeraDenovo*. We then merged the resulting feature tables from each study and merged the representative ESVs.

Taxonomic assignment was performed with a naive Bayesian classifier against the SILVA ribosomal RNA gene database v128 (Desantis et al., 2006; Quast et al., 2013) using *assignTaxonomy* in DADA2, and species assignment was performed by an exact string-matching algorithm using *assignSpecies* in DADA2. Chloroplast, mitochondrial, and archaeal sequences were removed from the ESV tables and representative ESVs using these taxonomic assignments (Note: several archaeal sequences were retained in the representative sequences as outgroups for the alignment and phylogeny). The final dataset contained 126,381 unique ESVs and a total sequence count of 31,512,311. An alignment and phylogeny of the representative ESVs were simultaneously estimated using the Practical Alignment method in SATé and TrAnsitivity (PASTA; Mirarab et al., 2015) against the SILVA v128 reference 16S rRNA gene sequence alignment and tree.

Our pipeline for standardized processing of the V4 hypervariable 16S rRNA Illumina raw data into ESV table, representative ESVs, and taxonomy are detailed here: https://github.com/MicrobiomeStressProject/Frontiers_Microbiology_2018/blob/master/main.md.

### Downstream Analyses: Finding Ecological Patterns Hidden in ESVs Across Studies

For the downstream analyses, instead of rarefying to the lowest suitable sequencing depth across the entire merged dataset, which would have been 1,074 sequences, we opted for per-study rarefactions (levels reported in Figure 2) in order to retain as much microbiome data as possible, ensuring maximal quality of the subsequent analyses (alpha diversity, beta diversity, resistance, and phylofactorization).

For each study treatment, we used the *core-metrics-phylogenetic* command in QIIME2 (Caporaso et al., 2010) to estimate three indices of alpha diversity (observed ESVs, Shannon-Weiner, and Pielou's evenness) and one of beta-diversity (Bray-Curtis dissimilarity). We used the *betadisper* command in the Vegan package version 2.5-2 in R (Oksanen et al., 2018) to calculate the distance to group centroid from the Bray-Curtis distance matrix for each study treatment. To identify significant study treatment effects on alpha diversity and beta diversity, we ran within-study Kruskal-Wallis tests for differences between the treated microbiomes relative to undisturbed control samples.

To conduct the meta-analysis, we calculated the proportional changes in treated samples relative to control samples for each study treatment (e.g., 25% decrease in observed ESVs or 15% increase in Pielou's evenness after diesel exposure in soil). Study-by-study treatment effect sizes were combined for each diversity metric and used to examine impacts of stressors to address our four objectives: (1) impact of warming on soil microbiomes (*n* = 79 samples), (2) the impact of a range of stressors in a soil environment (*n* = 325 samples), (3) the impact of copper in a variety of environments (*n* = 102 samples), and (4) overall impacts of stressors on microbiomes (*n* = 369 exposed microbiomes). We used a one-sample *t*-test of the distribution of effect sizes with μ_o_ = 0 to test for significance. The mean and confidence intervals for each categorical set of effect sizes were displayed as forest plots, along with the individual by study treatment results displayed in boxplots, each with corresponding symbols of significance (*p* < 0.05). For each study treatment, we estimated community resistance as the average pairwise similarity [1- Bray-Curtis dissimilarity] between the control microbiomes and the treated microbiomes (De Vries and Shade, 2013). Significant positive or negative correlations were identified on individual study treatments by examining the relationship between resistance and each alpha diversity index using linear models, and non-linear model fits for the overall relationship across all studies.

Finally, to identify ESVs and lineages responding to stressors, we performed phylofactorization (Phylofactor; Washburne et al., 2017) on just the subset of studies examining copper contamination (Objective 3). We acknowledge that there are a plethora of algorithms enabling differential abundance analysis for identifying taxa and/or clade responses to disturbance (Gloor et al., 2017; Weiss et al., 2017). We performed ANCOM analysis (Mandal et al., 2015) on each individual study treatment, along with Phylofactor, but decided to present just the Phylofactor results here, as our datasets spanned a wide range of environments (Figure 2), with very little overlap in ESVs among them. A central advantage of phylofactorization is the ability to detect similar shifts in microbial clades among studies when mapped to the same phylogeny, even with minimal ESV overlap. For example, the microbiomes from two environments may have no overlap in ESVs yet may still show overlap in clade membership (e.g., different microbial taxa identified as Actinobacteria). Even in the absence of ESV overlap among studies, if all members of a clade respond negatively to a stressor, this response will be apparent in the phylofactorization result. For computational ease and simplicity, we focused phylofactorization on the most prevalent members of microbial communities, defined as ESVs present in at least 20% of the microbiomes in a given study. Because the studies varied in both absolute copper treatment and actual copper exposure (i.e., difference in environmental retention of copper and bioavailability), certain studies with larger effect sizes would washout any phylofactor patterns of studies with more subtle responses. Consequently, to control for different effect sizes across studies, we inferred the phylogenetic factors, hereafter “phylofactor(s),” within each individual study and then mapped these phylofactors to a common phylogeny to identify overlap in clades among the copper stressor studies. Additionally, phylofactor is suitable for our dataset because it has no requirements for choosing a reference frame (i.e., non-responding microorganisms) to determine changes in microbial abundance in response to a disturbance. Phylogenetic factors were chosen as edges maximizing the F-statistic from least squares regression predicting isometric log-ratio (ILR) abundances with treatment. The number of significant factors was determined using Holm's sequentially-rejective multiple test procedure (Holm, 1979) with a 10% family-wise error rate cutoff.

## Results and Discussion

### Results of the Literature Search Reveals Thousands of Studies on Biotic and Abiotic Stressors in Host-Associated and Free-Living Environments

Our keyword search (restricted to dates between 2010 and April 2018) identified 12,687 published microbiome studies with 43% of the publications referring to at least one stressor keyword (*n* = 5,480, i.e., potential studies for the Microbiome Stress Project Database; Table S2). The bibliometric network of the journals that published these microbiome stress studies shows that this collective research spans a wide variety of disciplines, including medicine, ecology, ecotoxicology, food science, soil science, and plant sciences (Figure S1).

**Figure 3:**
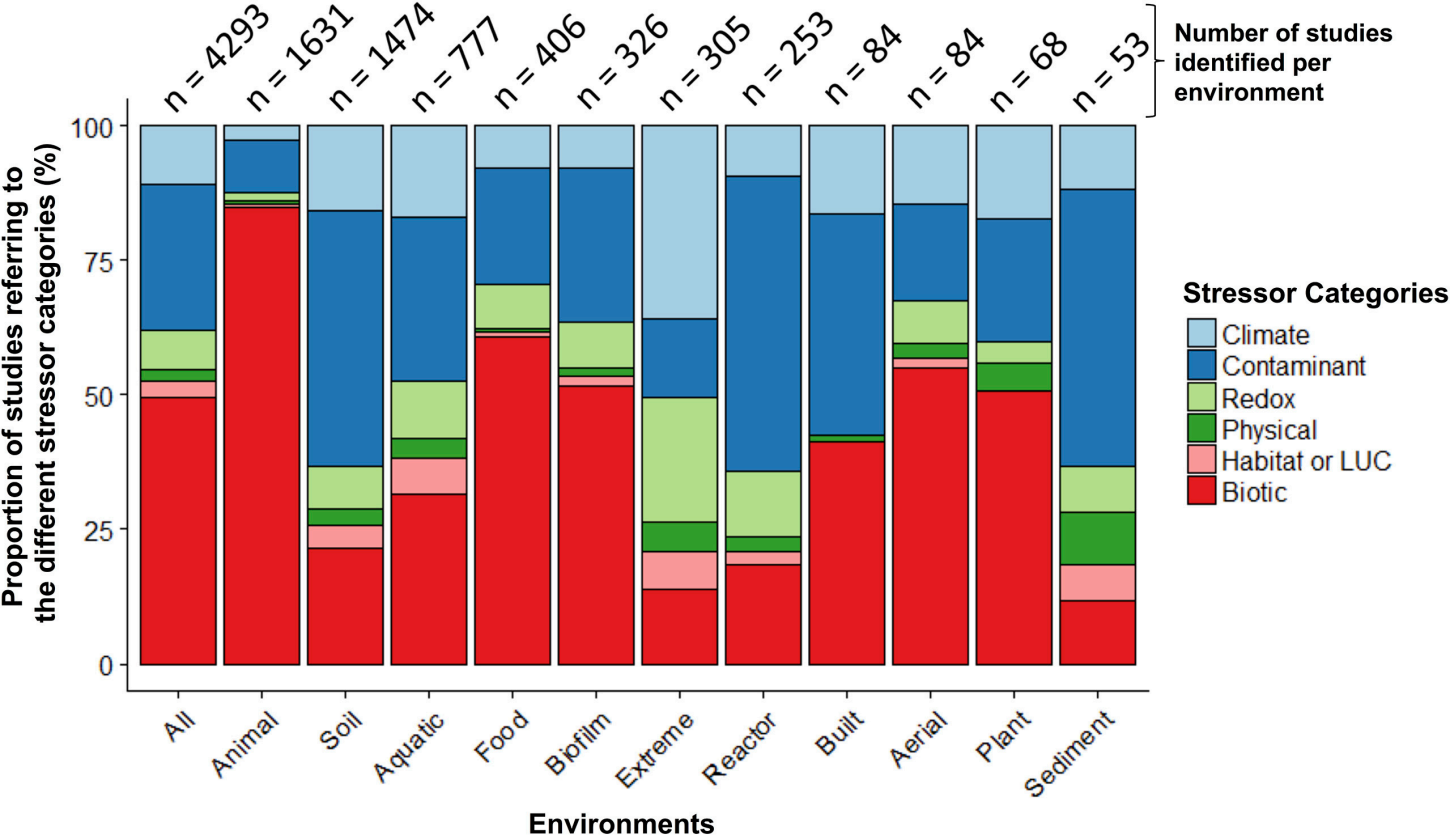
Relative proportion of microbiome stressor studies within different environments. The number of studies within each environment is at the top of each bar. The “Animal” environment includes human-microbiomes, and “LUC” stands for land use change.

When examining the stressors keywords cited in these 5,480 papers (no environment keywords included in this search), more than half of the studies (55%) referred to keywords categorized as “biotic” stressors (*n* = 3,306; e.g., pathogens, invasive species, herbivory, predation; Table S2). Of the remaining stressor categories, “contaminants” had the largest share (e.g., inorganic and organic pollutants; 27%) and “physical” had the smallest (e.g., fire, UV radiation; 2%). When examined within a co-occurrence network, stressor terms within studies clustered primarily into two separate groups—one dominated by global change (e.g., climate change) or ecotoxicology stressors (e.g., contamination), and the other by human health (e.g., disease, obesity; Figure S2). Pathogens occupied a central position within the network, highlighting the common interest shared by environmental and medical fields for these stressors. When microbiome studies were separated by environment (*n* = 4,293 when all environmental, stressor, and microbiome keywords combined; Figure 3; Table S2), the top three categories were: animal-associated (including human microbiomes, 38%), soil (34%), and aquatic ecosystems (18%). The three least referenced environment categories were: sediment, aerial, and plant-associated microbiomes, which were only referenced in a total of 5% of the studies.

Of the subset of 150 studies randomly screened from the 5,480 full set of studies in our literature search, 96% of the screened studies performed amplicon sequencing, using primarily Illumina (59%) and 454 pyrosequencing (31%) platforms and only 4% performed shotgun metagenomic sequencing. 89% of the studies sequenced the 16S rRNA gene to study the diversity of archaea and bacteria. Of the 16S rRNA gene studies, the V4 hypervariable region was the most frequently sequenced (76% of studies using primer sets: V3-V4, V4, V3-V5, & V4-V5). The next most common region was V3 (39% of studies using primer sets V1-V3, V2-V3, V3, V3-V4, V3-V5). Hence, the most common combination of sequencing platform and hypervariable region of the 16S rRNA gene studied was Illumina and the V4 region, respectively, which represented 57% of the 150 studies. Independent of those two classifications across all sequencing platforms and targeted genes, only 48 studies (64% of 150) had raw sequencing data available in public repositories (e.g., Sequence Read Archive) or via direct contact. Of the studies targeting the V4 hypervariable region of the 16S rRNA gene with Illumina sequencing, 69% had raw sequence data available in public repositories. Based on this literature search, for now we focus the Microbiome Stress Project Database and pilot study on the most common primer and sequencing platform combination (V4 region of 16S rRNA gene; Illumina MiSeq), with the pilot study results presented in section Results of the Pilot Study.

Moreover, while screening each individual study, we encountered many issues associated with unclear or incorrect meta-data, making these datasets unusable. The accessibility of both raw data and meta-data is absolutely crucial for integrating studies into larger meta-analyses like the Microbiome Stress Project. Our results clearly indicate that the microbiome research community still needs to commit to generating data that is open and accessible for reproducibility and synthesis.

In preparation for the full Microbiome Stress Project Database, we are also targeting unpublished microbiome studies to minimize publication biases, where published studies are more likely to have significant results. Including unpublished datasets, in addition to the published set, is crucial for minimizing publication bias for our meta-analyses. We have launched an online campaign using social media (Twitter: @MicrobialStress) and our website (microbiomestressproject.weebly.com) to solicit authors to contribute their unpublished datasets to our effort. In addition to contributing to the growing database, researchers will also be able to see how their microbial studies fit alongside comparable studies, or among studies of the same environment or stressor.

### Results of the Pilot Study

Among the 606 microbiomes, the average read depth was 39,761 sequences per sample (±38,902), resulting in 126,381 total merged ESVs (Figure 2), with only 2.2% (2882 ESVs) overlap among studies. The average rarefaction was 30,243 ESVs, and the actual rarefaction values for each study are presented in Figure 2. Despite large differences in per-study sequencing depth and in the number of ESVs observed, the two values were not correlated (*r*^2^ = 0.018).

#### Stressors Impact on Community Diversity and Resistance of Microbiomes

**Figure 4:**
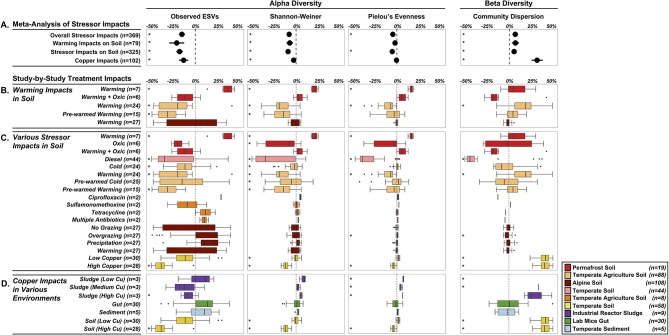
Shifts in alpha diversity (center three) and beta diversity (right panel) of treated microbiomes relative to control microbiomes. **(A)** Meta-Analysis of Stressor Impacts: forest plots summarizing the impact of stressors on community diversity across all microbiomes and the three subcategories. Each diversity metric was standardized by using proportional changes relative to undisturbed microbiomes. Study-by-Study Treatment Impacts **(B–D)** of the subcategories (colored by study-specific assignments—see Figure 2): boxplots summarizing the effects of individual study conditions on microbiomes relative to controls. **(B)** specific effects of elevated temperature on soil microbiomes, **(C)** impacts of 14 different stressors on soil microbiomes, and **(D)** copper impacts in four environments. Stars (^*^) to the left of each condition represent a significant effect *P* < 0.05. A box is missing for Sludge (Low copper) because the shift in community dispersion was 1.2-fold higher than the controls (*n* = 3).

Our first objective was to assess the specific impacts of warming on soil microbiomes. We found that warming treatments altered alpha diversity through decreased observed ESV richness (−21%, *P* < 0.0001) and Shannon-Weiner index (−7.2%, *P* = 0.0002), and no significant impacts on microbiome evenness (*P* < 0.0657) (Figure 4A). The overall decreased alpha diversity was primarily driven by one study focused on heat shock impacts in temperate agricultural soils that simulated extreme temperature conditions (+40°C, Jurburg et al., 2017). We acknowledge that this set of results is based on just three distinct studies (79 treated microbiomes), with substantial variation in the site-level characteristics as well as differences in the warming treatments, and as a proof-of-concept analysis, these trends should not be generalized. In trying to explain differential responses of diversity to warming, prior work in macro-ecosystems suggests a variety of diversity responses to disturbance, including non-monotonic responses (Mackey and Currie, 2001). Species often live in environmental optima, suggesting that moving in either direction from that optimum will result in a decrease in that species abundance (Holt, 2009). These unimodal patterns in niche space likely also give rise to unimodal diversity-disturbance relationships, if a large enough range of an environmental gradient is explored. This might explain the contrasted response of permafrost soil microbiomes to elevated temperature (+2C; Ernakovich et al., 2017), with increased alpha diversity across all indices (Observed ESVs: +34.5%, Shannon-Weiner: +21%, Pielou's Evenness: +16.2%, *n* = 7) (Figure 4B). Permafrost is frozen soil, so even slight increases in temperature results in drastic changes to the environment conditions. Thawing permafrost could potentially explain the increased alpha diversity, by “awakening” many microorganisms that are encysted, or otherwise metabolically inactive, in the frozen conditions (Makhalanyane et al., 2016; Wurzbacher et al., 2017) Alternatively, increased alpha diversity can also result from the growth of rare microbial taxa (below detection limit in controls) that rapidly respond to the warming disturbance (Shade et al., 2012; Coveley et al., 2015).

While warming decreased alpha diversity overall, community dispersion increased by 7% relative to control community dispersion (*P* = 0.0010), driven primarily by one significant increase from the same study examining heat-shocking temperate agriculture soils. Increases in microbial community dispersion have been observed in prior diversity-disturbance experiments (Gibbons et al., 2016), and may reflect a flattening of ecological selection as the environment becomes more heterogeneous. Additionally, this result could be explained by the fact that warming, and disturbances more generally, have stochastic rather than deterministic effects on microbiome composition leading to an increased dispersion compared to controls (i.e., the *Anna Karenina principle*, Zaneveld et al., 2017). Despite significant overall impacts of warming on soil microbial diversity, there was high variability in responses for each study treatment, which highlights the need for integrating more independent studies into the Microbiome Stress Project Database. This will increase the statistical power in these objectives, thereby generating reliable insights on how soil microbiomes respond to specific stressors like warming.

In our second objective, we expanded the analysis of soil microbiome responses to a wide range of stressors, which also resulted in decreased alpha diversity and increased community dispersion. Observed ESVs decreased by 17.7% (*P* < 0.0001), Shannon-Weiner by 9.1% (*P* < 0.0001), and Pielou's evenness decreased by 5.3% (*P* < 0.0001) across all soil microbiomes studies (Figure 4A), while community dispersion increased by 5.4% relative to control microbiomes (*P* = 0.0006).

Similar to the study specific impacts of warming, there was high variability in the soil microbiome responses to the range of stressors as well (Figure 4C). The studies testing the effects of warming, diesel, and copper primarily decreased across all alpha diversity metrics. Warming and copper imposed significant increases in community dispersion, consistent with the meta-analysis trend. In contrast, the diesel and overgrazing stressors resulted in significant decreases in community dispersion, which suggests that these stressors may strengthen ecological selection and lead to more deterministic shifts in composition (Zaneveld et al., 2017). Here, we only selected 12 stressors for this pilot study, but we already have 27 different stressors in the database, with multiple studies available per stressor. In future analyses, covering more soil systems and stressor exposures will allow us to validate the effects observed on alpha and beta-diversity in the pilot study and uncover the abiotic and biotic factors driving soil microbiome responses to disturbed conditions.

**Figure 5:**
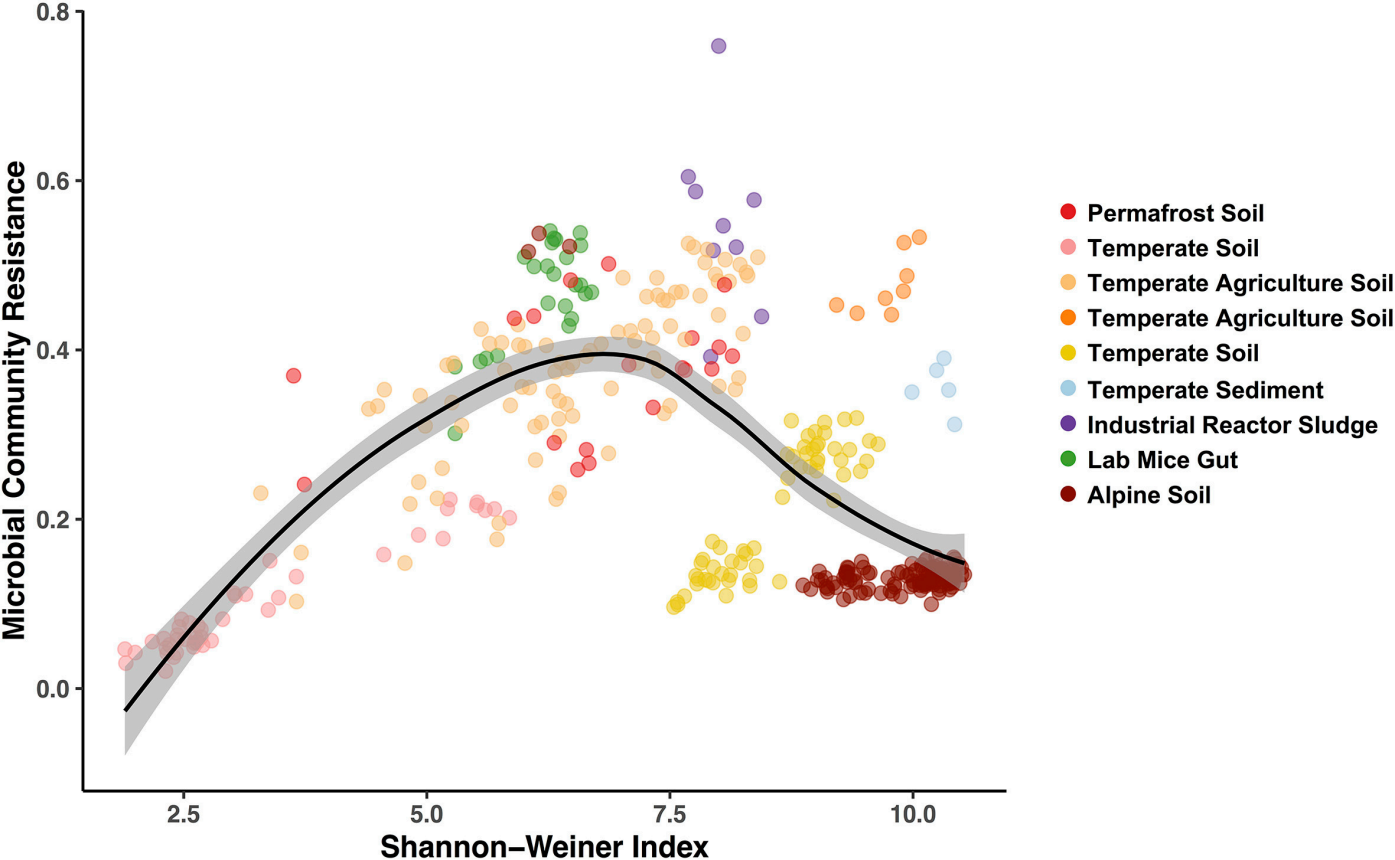
Hump-shaped relationship between microbiome community resistance to stressors and alpha diversity (Shannon-Weiner) of all treated microbiomes included in the pilot study. Data points represent a stressor treated microbiome (*n* = 369) and are colored by each study environment.

Our third objective was to evaluate the effects of an individual stressor across multiple environments. We examined copper contamination on 102 samples across the four environments (soil, sludge, gut, and sediment), and found similar decreases in alpha diversity: a 13.1% decrease in observed ESVs (*P* < 0.0001) and a 2.8% decrease in Shannon-Weiner (*P* = 0.0002). Like the other objectives, there was substantial among-study variability in alpha diversity in response to copper (Figure 4D). The overall negative impacts were primarily driven by high copper exposure in soil and sludge, while low copper treatments in soil and sludge and the copper treatments in gut and sediment were not significantly impacted by copper addition (Figure 2B). There was no significant overall impact of copper on community evenness (*P* = 0.2248). Community dispersion was 31.5% greater in copper treated microbiomes than control microbiomes (*P* < 0.0001), however this was primarily driven by the two copper treatments in soil (Nunes et al., 2016). The high variability in response of alpha diversity and beta diversity (Figure 5) may be due in part to the differential intrinsic resistance capacity of the microbiomes (e.g., initial alpha diversity or community network structure before exposure). Additionally, the magnitude of the treatment disturbance between studies (Figure 2) and the ecological memory of antecedent exposure to copper or other stressors may explain this range of copper effects observed on diversity (Griffiths and Philippot, 2013; Azarbad et al., 2016). The concentration and mode of exposure to copper were very contrasted between the four different studies which may explain a large part of the differences among studies (Figure 2). However, a very high chronic copper exposure in a mouse gut (1 g/L) did not cause any change in diversity (Figure 4), which suggests that the absolute magnitude of treatment may still not reflect actual exposure level for the microbiome, as in this case copper may be absorbed or transformed by the host before gut microbiome exposure (Cholewinska et al., 2018). Additionally, different redox potentials between environments may also determine actual exposure and speciation of copper, influencing the toxicity and bioavailability of this metal for microorganisms (Fleming and Trevors, 1989; Giller et al., 1998). Stochastic death throughout the treated microbiomes might also explain these patterns, with many microbial taxa driven to local extinction under high copper, resulting in distinct community structure and hence high beta diversity. While these results are interesting, we acknowledge again here that this pilot study does not have the statistical power to reliably draw any generalizable insights of copper impacts across multiple environments. With multiple studies for each environment-copper comparison (similar to the warming in soils) in the full dataset, we will be able to repeat the analysis with more power, accounting for copper bioavailability and historical exposures among studies.

Our fourth objective to analyze all 369 treated microbiomes in the pilot study revealed an average reduction in alpha diversity and increase in beta diversity. Similar to the results presented on the three other objectives of the meta-analysis, the exposure to all stressors led to significant decreases in the alpha diversity: observed ESVs decreased by 15% (*P* < 0.0001), Shannon-Weiner alpha diversity decreased by 7.8% (*P* < 0.0001), and evenness decreased by 4.6% (*P* < 0.0001) (Figure 4A). Beta diversity was also significantly affected by stressors, with community dispersion increasing by 6.7% (*P* < 0.0001) across all studies. These findings show that the diversity patterns observed for soil warming, a range of stressors in soil and copper across multiple environments still hold when performing a global meta-analysis of microbiome responses. These first results suggest that very contrasted stressors in diverse ecosystems lead to similar consequences for microbial community diversity. If these trends are substantiated with the same analysis at a larger scale, more research will be needed to understand how these shifts in diversity affect host health and ecosystem function. Moreover, using the growing Microbiome Stress Project database, researchers will be able to identify knowledge gaps and target research efforts for specific stressors or environments.

Finally, we used the Bray-Curtis similarities to estimate community resistance to stressors and explored potential correlations with the three alpha diversity indices (Figure 5; Table S3). We expected to see a positive correlation between alpha diversity and the microbiomes capacity to resist change (Tilman and Downing, 1994) and to remain stable when exposed to a stressor (i.e., the *Insurance Hypothesis*, Yachi and Loreau, 1999). However, when including all the exposed microbiomes, we primarily observed hump-shaped relationships between microbial community resistance and alpha diversity (Figure 5; relationship with Shannon-Weiner index). These results indicate that at low diversity levels (2 to 7.5 Shannon-Wiener index values, Figure 5), microbiome resistance is positively correlated to diversity but that this relationship becomes negative at higher diversity levels (7.5 to 10, Figure 5). These results suggest an alpha diversity optimum at which the microbiome resistance is maximal, which has been shown to exist in other microbial systems (Gibbons et al., 2016; Locey and Lennon, 2016). Elevated diversity can also be an indicator of a community in flux, where high dispersion rates in the environment increase the incidence of new microbial colonizers (Cadotte, 2006; Evans et al., 2017). In these systems, microbial populations may present a low residence time and a high community composition turnover (Locey and Lennon, 2016), which may drive in part the lower resistance observed at high diversity here. These preliminary results on diversity-stability relationships emphasize the need for a greater depth of studies for each stressor—environment combination to determine if these patterns are ubiquitous or occur under specific environmental conditions.

#### Phylofactorization of Microbiomes From Four Environments Exposed to Copper

**Figure 6:**
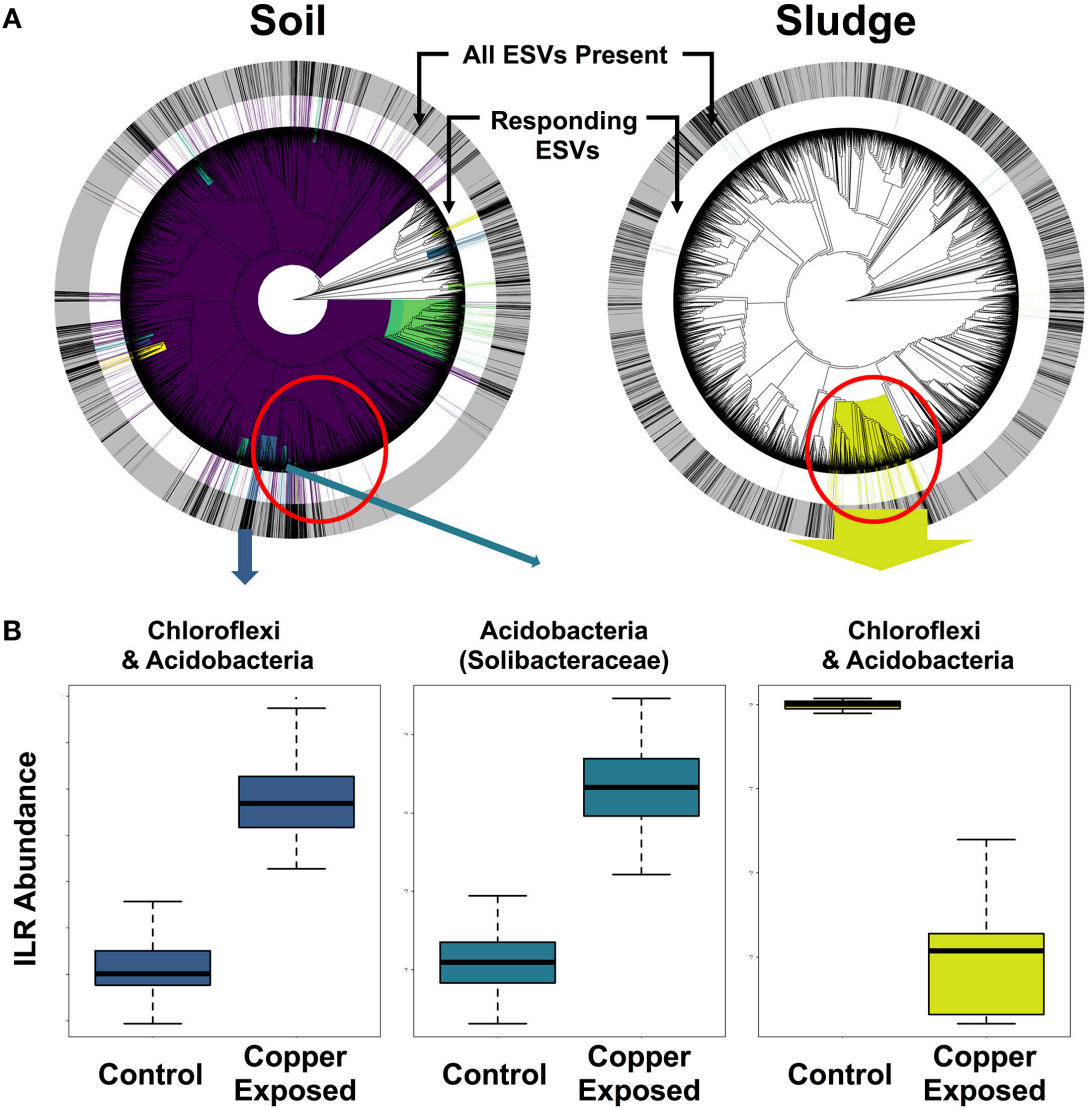
Phylofactorization of microbial clades responding to copper contamination in soil and sludge. **(A)** Shows the phylogenetic distribution of the phylofactors in both environments, with concentric rings displaying the distribution of all ESVs (outer gray ring) and copper-responding ESVs (inner white ring) in each environment. **(B)** ILR abundance shifts for *Chloroflexi* and *Acidobacteria*.

In order to isolate responding microbial ESVs and identify overlap in whole clades responding to copper contamination, we implemented Phylofactor. The four distinct environments exposed to copper had markedly different numbers of significant phylofactors (i.e., clades or terminal nodes responsive relative to the rest of the microbiome). The soil dataset had 780 phylofactors, covering 80% of the ESVs in the community, highlighting an extremely high community turnover in response to copper exposure (Figure 6A). In contrast, the gut microbiome had 20 phylofactors significantly responding to copper, the sludge had 17 phylofactors (Figure 6B), and the sediment had only five phylofactors containing 7 ESVs covering < 0.2% of the dataset. The higher number of phylofactors in soil and sludge are consistent with the large shifts in alpha and beta diversity observed in these two studies (Figure 4), and the large effect size in the soil dataset yielded a phylofactorization implicating nearly the entire phylogeny as responsive to copper treatment. One particularly large phylofactor in soil identified a lineage of 688 ESVs accounting for 47% of the community. While, there were no common ESVs among the copper responders across the environments, there was some overlap in responding clades found to be affected by copper exposure. In particular, a clade of 116 ESVs in the sludge dataset was close to four phylofactors in the soil dataset containing 20, 62, 10, and 9 ESVs, respectively (red circle in Figure 6). These phylofactors primarily contained uncultivated lineages annotated as members of *Acidobacteria, Solibacteraceae*, but also other *Acidobacteria* and Chloroflexi. These lineages are phylogenetically close, and in some cases nested clades but they interestingly presented opposite responses to copper treatment in the two environments (Figure 6). In the soil study, the responding community subsets of *Chloroflexi* and *Acidobacteria* were increased in the copper treated microbiomes, while they decreased in the sludge study (Figure 6). Other studies have demonstrated the sensitivity of both *Acidobacteria* and *Chloroflexi*, while Actinobacteria are generally tolerant to copper exposure in soils (Li et al., 2015) and in sludge (Sun et al., 2016). Due to the compositional nature of sequence-count data, it is impossible to determine how much of the effect is due to the populations of *Solibacteraceae*, for example, having opposite responses in the two environments and how much is due to the remaining community whose abundance shifts can confound directional inferences in compositional data. Rather than yielding clear inferences about potential bioindicator lineages in the current state, phylofactorization yielded more insight about future research on the difficulty of comparing results across microbiome studies, and the opportunities of datasets like the Microbiome Stress Project may yield to test and improve statistical tools. In addition to improving computational tools, it is important for researchers to improve quantification of absolute abundances by running qPCR or by including DNA spike-ins prior to sequencing to limit our reliance to relative abundance data. Concomitantly, future experimental work could directly target the microbial clades identified as responders to better understand their responses to individual stressor–environment scenarios and validate the inferences made in the amplicon sequencing meta-analyses.

### Concluding Remarks

In this article, we presented the objectives, methodological framework and bibliographic landscape covered by the Microbiome Stress Project and used our pilot study as a proof-of-concept meta-analysis to demonstrate the potential of this database. With thousands of studies available on the impact of environmental stressors on microbial communities, the Microbiome Stress Project Database is well-poised to allow researchers to tackle key ecological issues regarding the resistance, resilience, and response of microbial communities following exposure to environmental stressors. This project will generate a wealth of information on the natural history of microbial taxa, especially on their stressor tolerance and important life-history strategies for surviving in fluctuating environments. The full database will enable the identification of indicator taxa and clades to specific or multiple stressors that could eventually be used for monitoring or microbiome engineering. The Microbiome Stress Project provides an ideal resource for developing and testing new statistical methods with comprehensive amplicon sequencing datasets. We anticipate that research into common responses of microbial communities to stressors will lead to better microbiome diagnostics, allowing researchers to make inferences that are robust to both subtle and large-scale changes in species composition across independent studies.

The aim of this project is to identify global patterns in microbiome responses to stressors, but we encourage the scientific community to use the database to examine their own research questions. Adding your studies to the Microbiome Stress Project Database is a valuable contribution to the research community and also provides valuable context for your own work. Finally, building the database will reveal knowledge gaps in terms of missing or low replication studies of specific stressors or particular environments. We hope that the Microbiome Stress Project will forge new interdisciplinary collaborations leading to important breakthroughs in our understanding of microbial communities' responses to environmental change and for improving our ability to engineer microbiomes for improved human and environmental health.

## Author Contributions

JR, MS, JB, and FM conceived the project. MS and JB collected the raw sequences meta-data and conducted the literature search. FM and JR processed the sequence data. FM, AW, and SG developed the downstream analyses. MS, JR, and JE developed the conceptual framework and background synthesis. JR, MS, JB, JE, SG, FM, and AW contributed equally to writing the manuscript.

### Conflict of Interest Statement

The authors declare that the research was conducted in the absence of any commercial or financial relationships that could be construed as a potential conflict of interest.
