# Supplementary material for: The Microbiome Stress Project: Toward a Global Meta-Analysis of Environmental Stressors and Their Effects on Microbial Communities
Source: Front Microbiol. 2019 Jan 10;9:3272. doi: 10.3389/fmicb.2018.03272 (PMC6335337; doi:10.3389/fmicb.2018.03272)
Supplement: Supplementary file 1 [file Data_Sheet_1.docx]

**SUPPLEMENTARY MATERIALS**


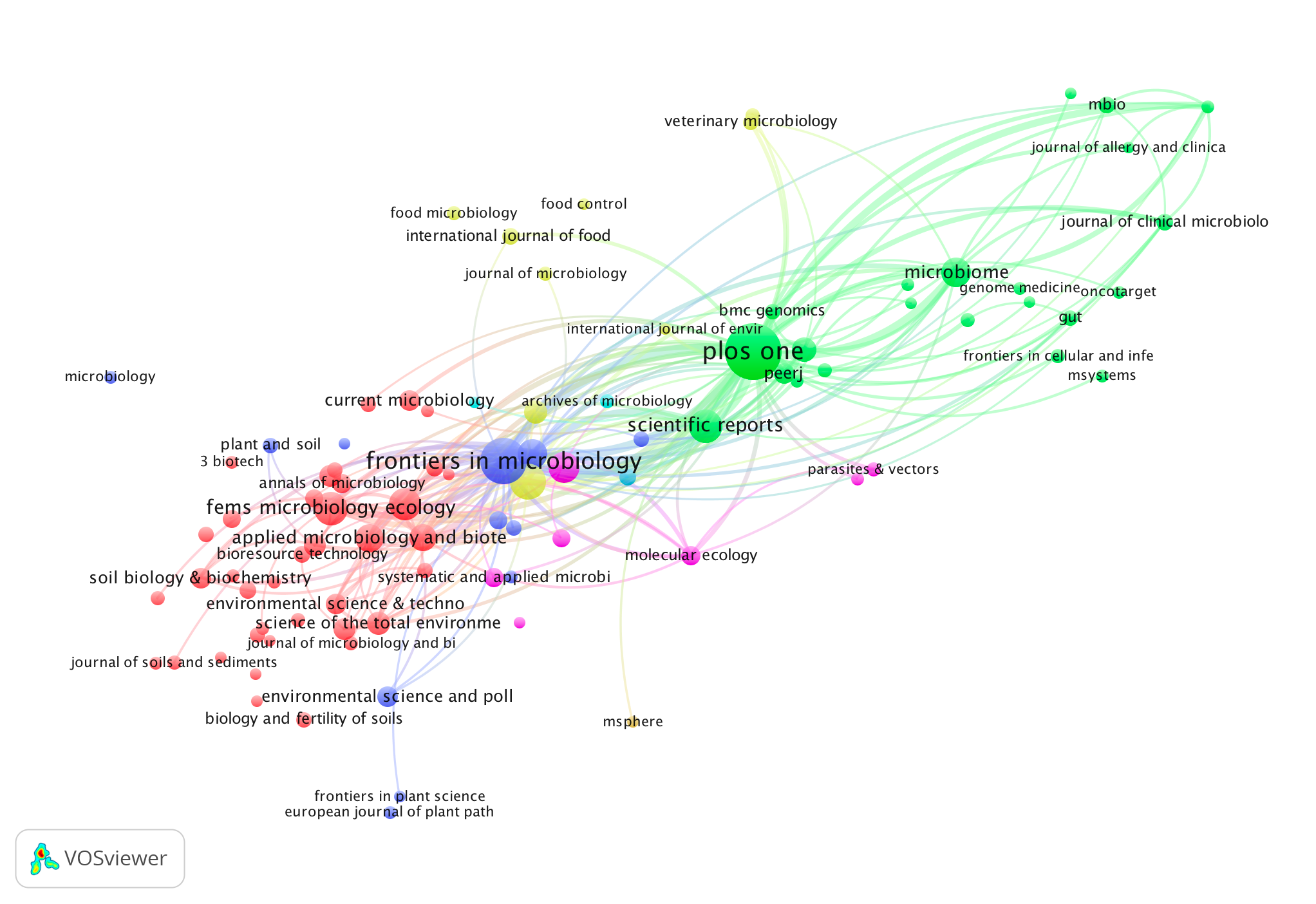


**Figure S1.** Network of the journals that published the studies identified in the literature search in Web of Science as research looking at the effects of stressors on microbiomes (n=5480 articles): Circle size is proportional to the number of articles published in each journal and the links/location of the circles represent co-citations among journals. Colors represent different clusters of journals that have a high level of interactions (co-citations), and line thickness is proportional to the number of shared citations between two journals (the first 200 strongest links are presented).

**
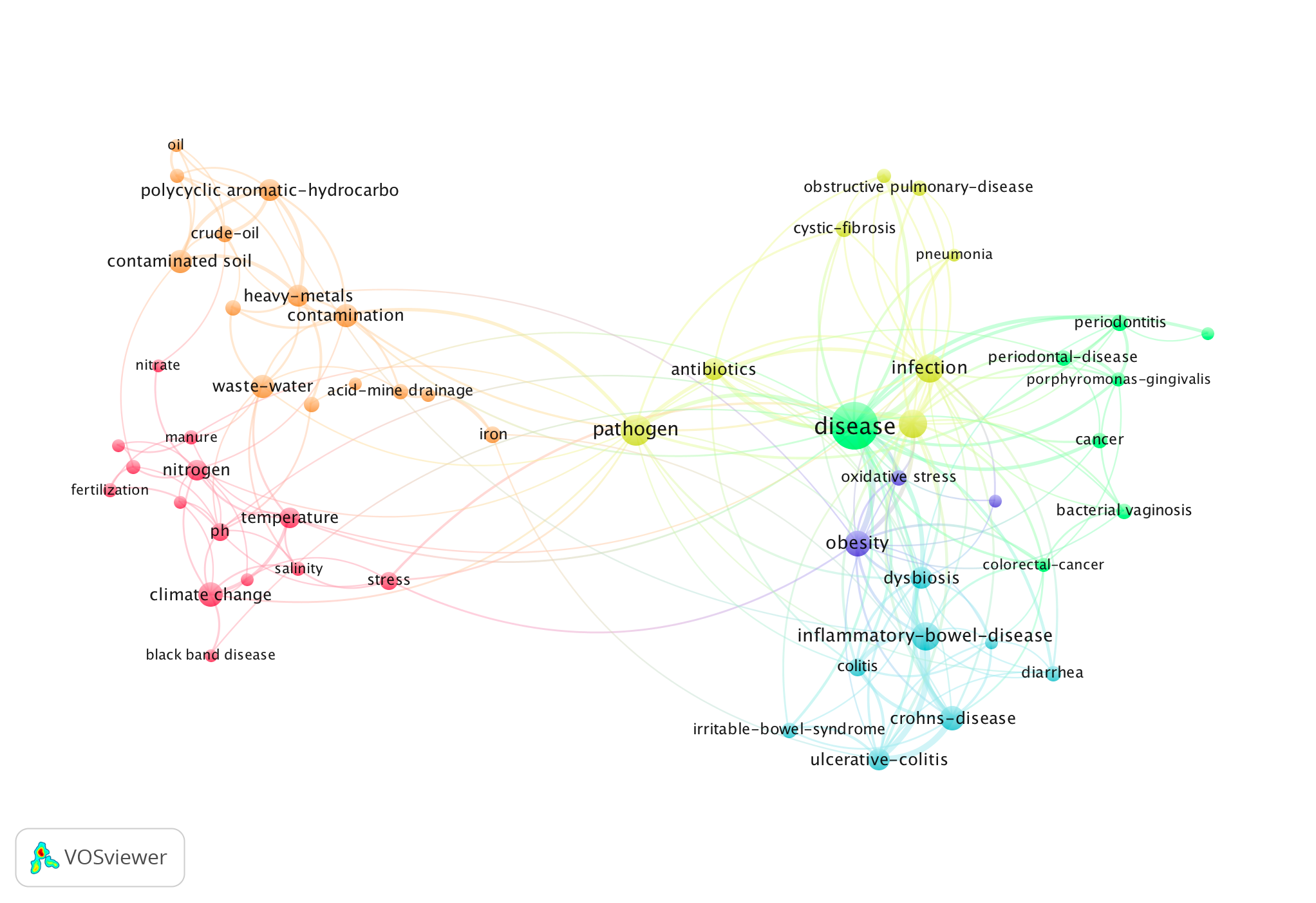
**

**Figure S2.** Network of co-occurrence between the most cited "stressor" terms in the articles identified in the Web of Science literature search as research looking at the effects of stressors on microbiomes. The line thickness is proportional to the number of co­occurrences between two terms (the first 200 strongest links are presented), and colors represent different clusters of terms that have a high co-occurrence.

**Table S1.** Comprehensive list of keywords used for the literature search in Web of Science to target the multidisciplinary set of microbiome studies looking at the effects of diverse stressors across multiple environments.

|  | Keywords & Format Used |
| --- | --- |
| GLOBAL |  |
| Microbiome | (microb* OR bacteri* OR archea*) AND (structure OR composition OR diversity) AND (sequencing OR metabarcoding OR amplicon) AND 16S |
| Stressors | stress* OR toxin* OR disturbance* OR pollut* OR contamin* OR ("global change") OR dysbiosis OR (“climate chang*”) OR (“climate-chang*”) OR (“global warming”) OR (“climate warming”) OR (“temperature change”) OR heat* OR frost* OR freeze-thaw* OR warming OR (“extreme temperature*”) OR drought* OR (“precipitation change*”) OR flood* OR storm* OR (“water stress”) OR (“extreme precipitation*”) OR (“extreme rainfall*”) OR ((elevated OR rising) AND (CO2 OR carbon dioxide)) OR (“CO2 enrichment”) OR ((pharmaceutic* OR (“synthetic organic chem*”) OR pesticid* OR (“personal care product*”) OR insecticide* OR neonicotinoid* OR (“polycyclic aromatic hydrocarbon*”) OR (“polychlorinated biphenyl*”) OR herbicide* OR disinfectant*) AND (pollution* OR contamin*)) OR (“organic contamin*”) OR (“inorganic contamin*”) OR (“metal pollut*”) OR (“metal contamin*”) OR (“mineral fertil*”) OR (((“heavy metal*”) OR mercury OR copper OR zinc OR arsenic OR selenium OR lead OR chromium OR chromium OR silver OR boron OR platinum OR nickel OR pharmaceutic* OR ("personal care product*") OR nanoparticle* OR nanomaterial* OR disinfectant* OR ("flame retardant*") OR neonicotinoid* OR antibiotic*) AND (pollution* OR contamin*)) OR ("emerging contamin*") OR ("endocrine disrupt*") OR (“nitrate pollut*”) OR (“ammon* pollut*”) OR (“nitrogen deposition”) OR (“excess nitrogen”) OR (“excess phosph*”) OR eutrophication OR (“nutrient pollution”) OR (“phosph* pollut*”) OR fertili* OR ("air pollution*") OR ("air pollutant*") OR ("particulate matter pollution*") OR hypoxia OR anoxia OR (“oxygen stress”) OR acidif* OR alkalin* OR (“acid rain”) OR ("alkaline mine drainage") OR ("acid mine drainage") OR (physical AND (disturbance* OR stress*)) OR wildfire* OR (fire* NOT “fire ant*”) OR (“light limitation”) OR (((“UV light”) OR (“ultra violet") OR UV-B OR ultraviolet) AND (radiation OR exposure)) OR (“light stress”) OR ("light pollution") OR (“salt stress*”) OR salinization OR (“saltwater intrusion*”) OR (“salinity stress”) OR (“sea level ris*”) OR (“sea-level ris*”) OR (habitat AND (loss OR degradation OR fragment*)) OR ("agricult* expans*") OR ("land use chang*") OR ("land-use chang*") OR ("land cover chang*") OR ("land-cover chang*") OR deforestation OR ("soil compaction") OR (“invasive species”) OR (“alien species”) OR (“invasive alien species”) OR ("invasive exotic species") OR ("exotic species") OR pathogen* OR disease* OR parasit* OR dysbiosis OR ("herbivore pressure*") OR (“herbivore attack*”) OR ("grazing pressure") OR ("over-grazing") OR ("trampling pressure*") OR (“predator attack*") OR ("predation pressure*") OR (“insect attack*”) OR (“insect outbreak*”) |
| STRESSORS |  |
| **Climate**: Temperature | (“temperature change”) OR heat* OR frost* OR freeze-thaw* OR warming OR (“extreme temperature*”) |
| **Climate**: Water Stress | drought* OR (“precipitation change*”) OR flood* OR storm* OR (“water stress”) OR (“extreme precipitation*”) OR (“extreme rainfall*”) |
| **Climate**: CO_2_ | ((elevated OR rising) AND (CO2 OR carbon dioxide)) OR (“CO2 enrichment”) |
| **Contaminant**: Organic | ((pharmaceutic* OR (“synthetic organic chem*”) OR pesticid* OR (“personal care product*”) OR insecticide* OR neonicotinoid* OR (“polycyclic aromatic hydrocarbon*”) OR (“polychlorinated biphenyl*”) OR herbicide* OR disinfectant*) AND (pollution* OR contamin*)) OR (“organic contamin*”) |
| **Contaminant**: Inorganic | (“inorganic contamin*”) OR (“metal pollut*”) OR (“metal contamin*”) OR (“mineral fertil*”) OR (((“heavy metal*”) OR mercury OR copper OR zinc OR arsenic OR selenium OR lead OR chromium OR chromium OR silver OR boron OR platinum OR nickel) AND (pollution* OR contamin*)) |
| **Contaminant**: Emergent | ((pharmaceutic* OR ("personal care product*") OR nanoparticle* OR nanomaterial* OR disinfectant* OR ("flame retardant*") OR neonicotinoid* OR antibiotic*) AND (pollution* OR contamin*)) OR (emerging contamin*) OR ("endocrine disrupt*") |
| **Contaminant**: Nutrients | (“nitrate pollut*”) OR (“ammon* pollut*”) OR (“nitrogen deposition”) OR (“excess nitrogen”) OR (“excess phosph*”) OR eutrophication OR (“nutrient pollution”) OR (“phosph* pollut*”) OR fertili* |
| **Contaminant**: Air | ("air pollution*") OR ("air pollutant*") OR ("particulate matter pollution*") |
| **Redox**: Oxygen | hypoxia OR anoxia OR (“oxygen stress”) |
| **Redox**: pH | acidif* OR alkalin* OR (“acid rain”) OR ("alkaline mine drainage") OR ("acid mine drainage") |
| **Physical**: Fire | wildfire* OR (fire* NOT “fire ant*”) |
| **Physical**: Light/UV | (“light limitation”) OR (((“UV light”) OR (“ultra violet") OR UV-B OR ultraviolet) AND (radiation OR exposure)) OR (“light stress”) OR ("light pollution") |
| **Physical**: Salt stress/Sea-level rise | (“salt stress*”) OR salinization OR (“saltwater intrusion*”) OR (“salinity stress”) OR (“sea level ris*”) OR (“sea-level ris*”) |
| **Habitat Loss/Land Use** | (habitat AND (loss OR degradation OR fragment*)) OR ("agricult* expans*") OR ("land use chang*") OR ("land-use chang*") OR ("land cover chang*") OR ("land-cover chang*") OR deforestation OR ("soil compaction") |
| **Biotic**: Invasive Species | (“invasive species”) OR (“alien species”) OR (“invasive alien species”) OR ("invasive exotic species") OR ("exotic species") |
| **Biotic**: Pathogens/Disease | pathogen* OR disease* OR parasit* OR dysbiosis |
| **Biotic**: Herbivory/Predation | ("herbivore pressure*") OR (“herbivore attack*”) OR ("grazing pressure") OR ("over-grazing") OR ("trampling pressure*") OR (“predator attack*") OR ("predation pressure*") OR (“insect attack*”) OR (“insect outbreak*”) |
| ENVIRONMENTS |  |
| Aquatic | aquatic OR marine OR seawater OR freshwater OR planktonic AND (microb* OR bacteri* OR archa*) |
| Soil | soil AND (microb* OR bacteri* OR archa*) |
| Animal-associated | gut OR intestinal OR fecal OR skin OR host-associated OR stool AND (microb* OR bacteri* OR archa*) |
| Sediment | ("sediment microb*") OR ("sediment bacteri*") OR ("sediment archa*") |
| Reactor | ("activated sludge") OR reactor OR ("wastewater treatment") OR ("anaerobic digest*") AND (microb* OR bacteri* OR archa*) |
| Aerial | airborne OR aerial OR aerosol AND (microb* OR bacteri* OR archa*) |
| Food | ("food industry") OR meat* OR poultry OR milk OR vegetable* OR cheese OR beverage OR fruit* OR bakery OR dairy OR seafood OR confectionery AND (microb* OR bacteri* OR archa*) |
| Plant-associated | rhizopher* OR phyllospher* OR epiphytic AND (microb* OR bacteri* OR archa*) |
| Biofilms | biofilm* OR biofouling OR periphyton |
| Built | (("built environment") OR indoor* OR building*) AND (microb* OR bacteri* OR archa*) |
| Extreme | ("hydrothermal vent") OR glacier* OR volcano* OR ("ocean trench") OR ("hot springs") OR geotherm* OR hypersalin* OR desert OR ice |

**Table S2.** Web of Science literature search results using keywords from Table S1 on April 16, 2018. Search results of all global “Microbiome” keywords yielded n=12,687 and all global “Stressor” keywords yielded n=3,295,611.

|  |  | Stressor Categories | | | | | |
| --- | --- | --- | --- | --- | --- | --- | --- |
| Environmental Categories | All Microbiome & Stressor keywords | Climate | Contaminant | Redox | Physical | Habitat Loss/Land Use | Biotic |
| Aquatic microbiome | 777 | 146 | 260 | 91 | 31 | 59 | 269 |
| Soil microbiome | 1474 | 246 | 747 | 124 | 47 | 66 | 337 |
| Animal-associated microbiome | 1631 | 47 | 163 | 25 | 9 | 11 | 1423 |
| Sediment microbiome | 53 | 7 | 31 | 5 | 6 | 4 | 7 |
| Reactor microbiome | 253 | 23 | 137 | 30 | 7 | 6 | 46 |
| Aerial microbiome | 84 | 15 | 38 | 0 | 1 | 0 | 38 |
| Food industry | 406 | 35 | 98 | 37 | 3 | 4 | 273 |
| Plant-associated microbiome | 68 | 13 | 17 | 3 | 4 | 0 | 38 |
| Biofilms | 326 | 28 | 100 | 30 | 5 | 7 | 181 |
| Built environment | 84 | 16 | 20 | 9 | 3 | 2 | 61 |
| Extreme environment | 305 | 109 | 45 | 71 | 16 | 22 | 42 |
| **With all environment keywords in the search** | **4293** | **500** | **1229** | **323** | **99** | **144** | **2238** |
| **No environment keywords, only stressor and microbiome** | **5480** | **593** | **1461** | **419** | **114** | **158** | **3036** |

**Table S3.** Correlation strength between resistance and each alpha diversity index. (*p-value codes: ***: < 0.001; **: 0.001-0.01; *: 0.01-0.05; +: 0.05-0.1; n.s.: > 0.1*)
